# In Vitro Bioactivity of a Recombinant Human Collagen Peptide in a Filler Biomimetic Skin Model

**DOI:** 10.1111/jocd.70592

**Published:** 2025-12-12

**Authors:** Chunyan He, Ranran Wang, Qiao Zhang, Yehong Wang, Nan Huang

**Affiliations:** ^1^ L'Oreal Research and Innovation Shanghai China

**Keywords:** filler, recombinant collagen peptide, regenerative medicine, skin model, tissue engineering

## Abstract

**Background:**

Recombinant human collagen peptides (rhCol peptides) have demonstrated considerable promise in various biomedical applications, including wound healing, dermatological treatments, aesthetic procedures, cosmetic formulations, and personal healthcare. Recently, their incorporation as a bioactive component in dermal filler products has gained significant attraction within the aesthetic and cosmetic fields.

**Aims:**

To elucidate the beneficial bio‐efficacy of rhCol peptides as a dermal filler, underlying mechanisms from both the cell and tissue levels were explored.

**Methods:**

The stimulatory effect of rhCol peptides on extracellular matrix (ECM) production was confirmed in 2D human fibroblast cultures at mRNA levels. Subsequently, its impact on skin rejuvenation was illustrated, utilizing a novel, in‐house developed 3D in vitro filler biomimetic skin model, tissue morphology, epidermal proliferation and differentiation, as well as dermal ECM deposition were investigated. Transcriptomic analysis further offered an in‐depth view of molecular pathways underlying the phenotypical change observed at cellular and tissue levels.

**Results:**

In the 2D cell model, matrix remodeling–related genes such as Collagen type I and III, Elastin, Fibrillin 1, Hyaluronic acid synthases 1, 2, and 3 were significantly upregulated upon treatment with rhCol III peptides. Its direct binding to the dermal scaffold was confirmed within a novel filler biomimetic in vitro skin model, providing compelling evidence of their beneficial effects on both epidermal and dermal compartments, embodied in improved epidermis regeneration (Ki67, COL17A1) and dermis remodeling (FBN1 and glycosaminoglycans). Furthermore, transcriptomic analysis provided valuable insights into the potential mechanisms of downregulated inflammation, senescence and apoptosis; upregulated integrin binding; and extracellular matrix formation resulted from the binding of rhCol III peptides to the dermal scaffold.

**Conclusions:**

These findings collectively support the pro‐ECM potential of such rhCol III peptides as a promising biomaterial for dermal filler, as well as potential cosmetic applications. Limited by the gap between in vitro mimetic model and real injection, further clinical trials should be taken to reconfirm the potential benefits.

## 1 Introduction

Collagen, a critical component of the extracellular matrix (ECM), encompasses diverse subtypes and intricate supramolecular fibrillar arrangements, contributing significantly to the mechanical and functional properties of tissues such as skeleton, cartilage, bone, tendon, and skin [1]. Pathological conditions and age‐related ECM alterations are frequently associated with collagen degradation and morphological changes [2, 3], inspiring therapeutic strategies focusing on collagen replenishment or modulating neocollagenesis to mitigate disease progression, alleviate symptoms, and improve overall health. The concept of matrikines, primarily encompassing proteolytic fragments of endogenous ECM proteins including collagen, has spurred the widespread use of orally administered animal‐derived collagen hydrolysates in the nutricosmetic market [4, 5, 6, 7]. Studies have reported that oral collagen peptide supplementation and intracutaneous injection of collagen enhance skin hydration and improve the dermal collagen network, supporting its application in cosmetic and aesthetic procedures, both in topical products and as injectable biomaterials [6, 8, 9]. However, the transient native skin benefits observed upon cessation of supplementation raise concerns regarding the bioavailability and absorption of these large molecular weight collagen peptides when applied orally or topically. Furthermore, potential immunological reactions pose a significant challenge for the utilization of animal‐derived collagen peptides in aesthetic procedures.

Recombinant human collagen (rhCol) peptides, generated through advanced biotechnology and fermentation engineering, present a compelling alternative. These peptides, possessing amino acid sequences identical to endogenous human collagen but with reduced molecular weight, offer improved bioavailability and skin absorption. The incorporation of functional motifs during sequence design further enhances their bioactivity. Certain collagen‐mimetic peptides have demonstrated the capacity for self‐assembly into triple‐helical structures resembling native collagen, exhibiting enhanced cell adhesion properties, promoting cytokine secretion, reducing platelet aggregation, and accelerating wound healing [10, 11, 12, 13, 14]. Studies utilizing human epidermal keratinocytes and dermal fibroblasts have partially unveiled the mechanisms underlying the bioactivity of collagen peptides, with implications on cell proliferation, migration, anti‐inflammatory and anti‐oxidant responses, and ECM production [15, 16, 17, 18]. Notably, in vivo studies employing topical serum formulations containing collagen peptides have demonstrated efficacy in mitigating photoaging, highlighting the involvement of TGF‐β/Smad pathway activation and inhibition of collagen degradation [19].

Injectable fillers constitute a cornerstone of aesthetic dermatology, employing diverse biomaterials such as collagen, poly‐L‐lactic acid (PLLA), polymethyl methacrylate (PMMA) microspheres, silicone, free or cross‐linked hyaluronic acid (HA) of varying particle sizes to achieve either immediate volumization and wrinkle reduction or sustained lifting effects [20, 21, 22, 23, 24]. Given their human origin, structural similarity to endogenous collagen, and notable in vitro bioactivity, rhCol peptides represent a promising addition to the filler landscape, offering potential advantages including biocompatibility, minimal risk of immunological reactions, enhanced skin quality, stimulated ECM remodeling, and accelerated wound healing [12, 25, 26, 27]. However, investigations elucidating the precise mechanisms of action of rhCol peptides following filler injection remain limited. Further research is needed to understand how these peptides integrate into the ECM scaffold, contribute to the matrikine pool, engage in receptor‐mediated signaling pathways, modulate dermal fibroblast activity, and influence epidermal function through dermo‐epidermal crosstalk.

While human biopsy samples from filler injection sites would provide ideal biological insights, their accessibility is limited due to the elective nature of these procedures. Full‐thickness three‐dimensional (3D) in vitro skin models, comprising both epidermal and dermal layers reconstructed from human keratinocytes and fibroblasts, demonstrate similar characteristics to native skin regarding structure, biomarker expression, and biological responses, and thus offer a valuable alternative for exploring filler bioactivity [28, 29]. However, the relatively thin dermis in these models presents technical challenges for conducting simulated filler injections with consistency. To overcome this limitation, pre‐mixing rhCol peptides into the collagen matrix during dermis construction could realize a surrogate solution for diffusing filler materials within the dermal compartment. Herein, we present an actual use‐case of such “filler‐mimetic” in vitro skin model enabling the investigation of skin morphology, biomarker expression, and transcriptomic profiles, to elucidate the intricate bio‐mechanisms underlying rhCol peptide activity. This in vitro pre‐mixing model, limited by its constrained cellular composition and the absence of a dermal microvascular system, cannot fully reproduce the micro‐distribution observed in a true in vivo injection. Nevertheless, it has provided extensive investigative possibilities and shed crucial light for subsequent clinical reconfirmation.

## 2 Materials and Methods

### 2.1 Reagents

RhCol III peptides, a rhCOL peptide with genome sequence identical to Gly483‐Pro512 of human COL3A1 [10] were obtained from Shanxi Jinbo Biopharmaceutical Ltd. Antibodies used in immunofluorescence and immunohistochemistry staining were purchased from Proteintech (anti Transglutaminase 1, 12912‐3‐AP, Rosemont, IL, USA), Dako (anti Ki67, M7240, Carpinteria, CA, USA), Abcam (anti Coll XVII, M7240, Shanghai, China), Southern Biotech (anti Fibrillin 1, 1405‐01, Birmingham, AL, USA), Invitrogen (Donkey anti‐mouse 488, A‐21202, Shanghai, China), Solarbio (Alcian Blue stain kit, pH 2.5, G1560, Beijing, China). Cell culture medium, as well as fetal bovine serum (FBS), antibiotics, and Dulbecco's phosphate buffer saline (DPBS) were acquired from Gibco (Thermo Fisher, Waltham, MA). Vitamin C was purchased from Sigma (A8960).

### 2.2 Human Dermal Fibroblast Culture

Normal human dermal fibroblasts were purchased from BeiNaTronics and grown in DMEM, containing 10% FBS, at 37°C and 5% CO_2_. Cells at passage 7 were seeded into 6‐well plates at a density of 1 × 10^6^ cells/well for RNA analysis. The cells were grown for 24 h in complete culture medium (DMEM with 10% FBS) and then treated for 24 h in maintenance medium (DMEM with 1% FBS). Cells of the negative control group (NT) were cultured in maintenance medium. Vitamin C (Vc) at 200 μM was added in the positive control group (PC) with fresh maintenance medium. RhCol III peptide was administered at the concentrations of 100 and 33 μg/mL. Following the treatment, the cells were incubated for 24 h before being harvested, with triplicates per group.

### 2.3 Quantification of mRNA Transcription Using RT‐qPCR

Human dermal fibroblasts were collected for RT‐qPCR analysis. Primer design: Internal reference fragment: 18 s‐112 bp, Objective fragment: COL‐I‐164 bp, COL‐III‐140 bp, Elastin‐140 bp, FBN1‐160 bp, HAS1‐176 bp, HAS2‐180 bp, HAS3‐150 bp. Sequence details of all the primers are listed in Table 1. Single peak melt curves and cycling curves are provided in Data S7.

**TABLE 1:** Primer details for qPCR.

Name	Sequence‐Forward	Sequence‐Reverse	Efficiency
COL1A1	CCTGGATGCCATCAAAGTCT	ACTGCAACTGGAATCCATCG	1.0
COL3A1	TTCAGTTTAGCTACGGCAATCC	GGCCTGATCCATGTATGCAA	1.0
ELN	TGAAGCCTCAAAGCTGGATT	AAGGTGGCTATTCCCAGTGT	1.0
FBN1	GTCAGCCCCAGTTTAATACAC	CCTGTTAGGTGGAAGAAAGC	1.0
HAS1	CCACTGTGTATCCTGCATCA	TGGAGGTGTACTTGGTAGCAT	1.0
HAS2	TTGTTGGAACGTTGCTCTAT	ACAGCCCCTAGAGGAACTAA	1.0
HAS3	GGGAAAGGTATGGCAGTAGA	CAGGTCCCAGTTCACATGTA	1.0
18s rRNA	CCTGGATACCGCAGCTAGGA	GCGGCGCAATACGAATGCCCC	1.0

Total cellular RNA was isolated using Trizol and chloroform. Subsequently, cDNA synthesis was performed on 1 μg of RNA (in 15 μL RNase‐free water) using the iScript cDNA synthesis kit in a 20 μL reaction volume in a programmable thermal controller for 5 min at 25°C, 30 min at 42°C, and 5 min at 85°C. The cDNA was diluted 20 times in RNase‐free water and used as a template for qPCR amplification. The reaction was performed in a total volume of 20 μL containing 5 μL cDNA, 1 μL primers, 10 μL 2× SYBR Green super mix, and 4 μL dH_2_O. After incubation for 5 min at 95°C, amplification was carried out for 40 cycles of 15 s at 95°C and 32 s at 60°C. The purity of the amplified product was assessed using a melt curve analysis. The melting temperature was measured by increasing the temperature from 60°C to 95°C with 0.5°C increments every 10 s to indicate sample purity.

### 2.4 Reconstructed Full‐Thickness Skin Model

Dermal equivalents (lattices) were prepared using human fibroblasts embedded into a bovine type I collagen gel. After 4 days of contraction, human keratinocytes were seeded on top of the dermal equivalents. Cultures were kept submerged for 7 days and then raised at the air‐liquid interface for one more week to produce a stratified and differentiated epidermis.

The reconstructed full‐thickness skin model was fabricated based on established protocols [28]. Human fibroblasts were cultured in DMEM (Gibco 10569, Thermo Fisher, Waltham, MA, USA) containing 10% FBS (Hyclone SH30084, Thermo Fisher, Waltham, MA, USA) before 3D culture. Dermal equivalents (lattices) were prepared using 7 mL of a bovine type I collagen gel (Symatese, France) and 10^6^ fibroblasts mixture. After 4 days of contraction, normal human keratinocytes were seeded on top of dermal equivalents. Cultures were kept submerged for 7 days and then raised at the air–liquid interface for one more week to produce a stratified and differentiated epidermis.

### 2.5 3D In Vitro Filler Mimetic Skin Model

On lattice fabrication day, rhCol III peptides were mixed within lattice medium to fabricate dermal equivalents. The subsequent steps were the same as the standard procedure for full‐thickness skin model production.

### 2.6 Histology

After 22 days of treatment, skin models were punched and fixed in formalin for histology. Paraffin sections (5 μm) were stained with hematoxylin–eosin–saffron (HES) under standard procedures.

### 2.7 FITC‐rhCol III Peptides Labeling

RhCol III peptides at 1 mg/mL in PBS were adjusted to pH 8.5 before reacting with FITC probes for 4 h avoiding light at RT. Dialysis was followed to remove free FITC probes. A final FITC‐rhCol III peptides solution of 1 mg/mL, with calculated dye: protein (F/P) of 3.62, was stored under −20°C for further use.

### 2.8 Multiphoton and Confocal Observation

Dermal equivalent with FITC‐rhCol III peptides was fabricated under a volume ratio of 1:200 (1 mg/mL FITC rhCol III peptide/lattice medium). Four days after lattice contraction, the whole tissue was taken into observation of collagen scaffold and labeled rhCol peptide under a multiphoton and confocal microscope. The collagen scaffold signal was detected using a Nikon A1R multiphoton microscope via second harmonic generation (SHG) upon excitation with a Mai Tai femtosecond laser (760 nm). Separately, the fluorescence signal from the labeled rhCol peptide was collected using a Nikon A1 confocal microscope with a 488 nm laser. Images from both confocal and multiphoton were acquired from the same Regions of Interest (ROIs)and subsequently processed with ImageJ.

### 2.9 Immunofluorescence Staining

On day 22, skin models were punched into 10 mm‐diameter specimens and immediately embedded in tissue freezing medium (Leica, 14020108926, Wetzlar, Germany) in steel containers, snap‐frozen in liquid nitrogen, and then stored at −80°C for immunofluorescence. Tissue sections with 7 μm thickness were prepared using a cryostat (Leica) and collected on glass slides. Samples were stored at −80°C before immunofluorescence staining. For this, slides were fixed with ice‐cold acetone for 20 min and air‐dried for 30 min at RT. Tissue sections were rehydrated in PBS, then blocked in PBS containing 5% goat serum solution for 60 min. Primary antibodies were incubated for 2 h at RT. Slides were washed three times with PBS. Next, corresponding secondary antibodies were incubated for 1 h at RT followed by three washes in PBS. Afterwards, samples were further incubated with DAPI (Dojindo, D523, Tabaru, Japan) solution for 5 min followed by three washes in PBS. Finally, slides were mounted using mounting medium (DAKO, S3023, Glostrup, Denmark) and observed on an inverted Leica microscope. Antibody dilution was as follows: 1:50 for anti‐Fibrillin1, 1:200 for anti‐Col XVII.

### 2.10 Immunohistochemistry Staining

Samples of reconstructed skin were embedded in paraffin and cut into 5 μm thick sections. Paraffin sections were baked at 60°C for 30 min, then deparaffinized by placing them through xylene and serial alcohol for 32 min. Antigen retrieval was then achieved via autoclaving at 99°C for 20 min. The sections were preincubated with 3% hydrogen peroxide in distilled water for 5 min to block endogenous peroxidase activity, and then blocked in PBS containing 2.5% donkey serum (Jackson, JAC‐017‐000‐121) solution for 30 min. The slides were then washed in Tris‐buffered saline and incubated with primary antibodies at room temperature for 60 min. After incubation with primary antibodies, the samples were rinsed with Tris‐buffered saline and re‐incubated with a secondary antibody for 15 min at room temperature. Incubated samples with DAB for 5 min with DAB followed with hematoxylin staining for 10 s. Finally, the slides were dehydrated and sealed with neutral balsam. Antibody dilution was as follows: 1:100 for anti‐TGM1, 1:100 for anti‐Ki67.

### 2.11 Alcian Blue Staining

Samples of reconstructed skin were processed into 5 μm thick sections and deparaffinized as mentioned earlier. The sections were subsequently put into Alcian blue acidifying liquid for 3 min for acidification, then incubated in Alcian blue solution for 30 min followed by a water rinse. After that, nuclear fast red was applied to counterstain for 5 min, followed by a water rinse. The last step was dehydration and sealing with neutral balsam. Alcian Blue Stain Kit (pH 2.5, Solarbio, cat G1560) is used to stain sulfated acid mucopolysaccharides (hyaluronic acid [HA]) and other sulfated and carboxylated sialomucins (glycoproteins) deposited in the extracellular matrix.

### 2.12 RNA Extraction, Library Preparation, and Sequencing

Total RNA was extracted from the skin model using TRIzol Reagent according to the manufacturer's instructions (Magen). RNA samples were detected based on the A260/A280 absorbance ratio with a Nanodrop ND‐2000 system (Thermo Scientific, USA), and the RIN of RNA was determined by an Agilent Bioanalyzer 4150 system (Agilent Technologies, CA, USA). Only qualified samples will be used for library construction.

Paired‐end libraries were prepared using a VAHTS Universal V6 RNA‐seq Library Prep Kit for Illumina(Vazyme) following the manufacturer's instructions. The mRNA was purified from 1 μg total RNA using oligo (dT) magnetic beads followed by fragmentation carried out using divalent cations at elevated temperatures in ABclonal First Strand Synthesis Reaction Buffer. Subsequently, first‐strand cDNAs were synthesized with random hexamer primers and Reverse Transcriptase (RNaseH) using mRNA fragments as templates, followed by second‐strand cDNA synthesis using DNA polymerase I, RNAseH, buffer, and dNTPs. The synthesized double‐stranded cDNA fragments were then adapter‐ligated for preparation of the paired‐end library. Adaptor‐ligated cDNA was used for PCR amplification. PCR products were purified (AMPure XP system), and library quality was assessed on an Agilent Bioanalyzer 4150 system. Finally, the library preparations were sequenced on an Illumina Novaseq 6000 (or MGISEQ‐T7), and 150 bp paired‐end reads were generated. CRO, APPLIED PROTEIN TECHNOLOGY, conducted these processes.

### 2.13 RNA‐Seq Data Analysis

RNA‐seq raw data quality was assessed using Fastp (v0.23.2) for adaptor trimming and low‐quality read filtering. Following quality control, clean reads were aligned to the human reference genome (GRCh38) using HISAT2 (v2.2.1) with default parameters. The average total mapping rate was 97.91%. The resulting aligned BAM files were subsequently processed for gene‐level quantification using featureCounts (v2.0.3) based on the same Ensembl GRCh38 GTF annotation file. For transcript‐level assembly and quantification, StringTie (v2.2.1) was also applied in a reference‐guided mode to estimate transcript abundances (FPKM/TPM) and to reconstruct novel isoforms.

### 2.14 Differential Gene Analysis

DESeq2 R package [30] (version 1.34.0) was used to examine whether genes were differentially expressed between the treatment and control groups based on read count.

### 2.15 Pathway Enrichment Analysis

Differentially expressed genes were subjected to gene ontology and KEGG pathway enrichment analysis in DAVID with a *p* value < 0.05. We used gene sets of molecular pathways from the KEGG, Reactome, GO databases to compute pathways [31, 32].

### 2.16 Data Analysis

Three well replicates per treatment were used for qPCR analysis. For histology/IHC/IF staining, three tissue replicates per treatment were used. Within each replicate, at least three randomly chosen, non‐redundant Regions of Interest (ROIs) of constant dimension were selected to ensure representative sampling across the entire tissue section. The intensity of proteins in the ROIs was quantified using Image J. Brown‐Forsythe and Welch ANOVA tests were applied to all group comparisons with FDR (Benjamini–Hochberg) correction. *p* < 0.05 represented a statistical difference. Diagrams showing mean ± SEM were generated using GraphPad Prism 10.2.2(397) software (GraphPad Software Inc., La Jolla, CA, USA).

### 2.17 Data Availability

RNA‐Seq data have been deposited in the NCBI Sequenced Read Archive under the accession number SRR35847494.

## 3 Results

### 3.1 RhCol III Peptides Stimulated Extracellular Matrix Biomarker Expression in Normal Human Dermal Fibroblasts

**FIGURE 1:**
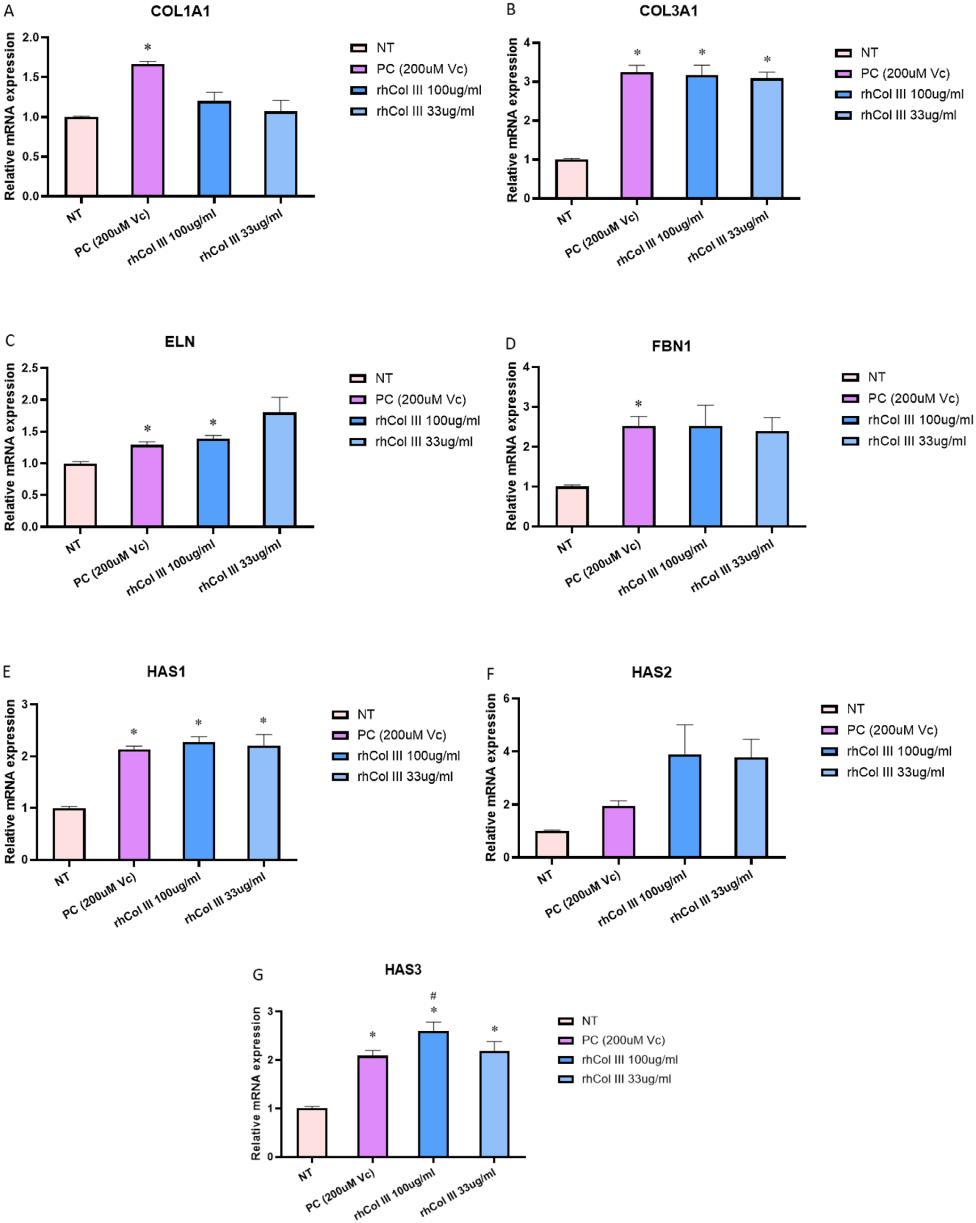
The effect of rhCol III peptides on extracellular matrix gene expression in normal human dermal fibroblast (NHDF) cultures. Gene expression was measured by qPCR in cell lysate of NHDF cultured in 6‐well plates and incubated in media plus rhCol III peptides at two concentrations (100, 33 μg/mL) or Vitamin C (200 μM) for 24 h. Quantification was performed with ΔΔCt normalized to 18s rRNA. Data were normalized to media control (NT) in each experiment and presented as mean ± SEM. *N* = 3. One‐way ANOVA was applied with FDR (Benjamini–Hochberg). *p < 0.05. # *p* < 0.05 when compared with PC group. Statistical details are included in Data S1.

The impact of rhCol III peptides on ECM‐related gene expression was assessed in human dermal fibroblasts following 24‐h exposure to two concentrations (100 and 33 μg/mL). Ascorbic acid (200 μM) served as a positive control. Genes listed in Table 1 were detected under the corresponding primer sequences. Compared with untreated controls, rhCol III peptides at both concentrations significantly upregulated mRNA expression of all examined ECM biomarkers (Figure 1). COL1A1 expression exhibited a modest increase, albeit less than 1.5‐fold without significance. Conversely, the expression levels of COL3A1, ELN, FBN1, HAS1‐2 were comparable to those observed with ascorbic acid treatment. Notably, HAS3 expression following treatment with 33 μg/mL rhCol III peptides, surpassed the levels induced by ascorbic acid.

### 3.2 FITC‐rhCol III Peptides Colocalize With Dermal Collagen Scaffold

**FIGURE 2:**
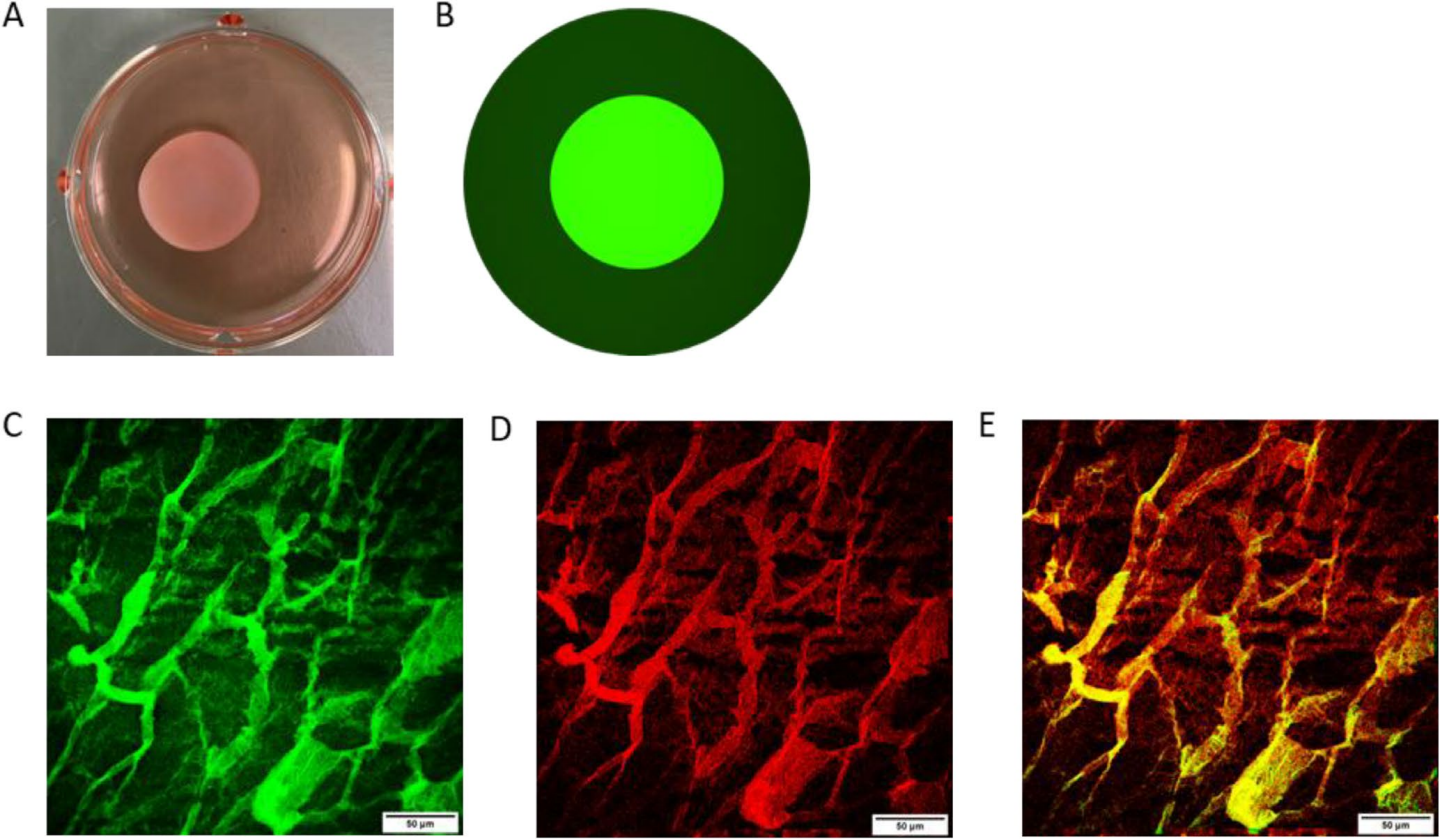
FITC‐rhCol III peptides deposited and bound with the scaffold of type I of collagen. (A) One lattice after 4 days of contraction. (B) Simulation of FITC intensity in lattice and the surrounding medium, which is based on fluorescent pictures. The volume ratio of FITC‐rhCol III peptides and lattice is 1:200. (C) Lattice image taken by Confocal microscope. The green signal represents FITC‐rhCol III peptides (laser wavelength: 488 nm). (D) Lattice image taken by Multiphoton microscope. The red signal represents the scaffold of type I Collagen (IR laser wavelength: 760 nm). Confocal and multiphoton images were acquired from the same ROIs. (E) Image merged. Field of view (C–E): 300 × 300 μm. Scale bar 50 μm. Software Nikon NIS‐Elements.

Before constructing the 3D in vitro filler mimetic skin model, a proof‐of‐concept experiment was conducted using fluorescein isothiocyanate (FITC)–labeled rhCol III peptides to ascertain the retention of injected filler within the dermal compartment. Premixing FITC‐rhCol III peptides with the collagen solution used for dermal lattice construction did not affect lattice contraction (Figure 2A). Under fluorescence microscopy, a strong FITC signal was observed within the contracted lattice, while a comparatively weaker signal was detected in the surrounding medium (Figure 2B). Confocal and multiphoton microscopy revealed co‐localization of the FITC signal (Figure 2C) and the collagen signal derived from multiphoton imaging throughout the dermal scaffold (Figure 2D). These findings confirm that premixed rhCol III peptides are not only retained within the dermal lattice but also associate with the dermal scaffold itself.

### 3.3 In Vitro Bioactivity of rhCol III Peptides Within 3D In Vitro Filler Mimetic Skin Model

**FIGURE 3:**
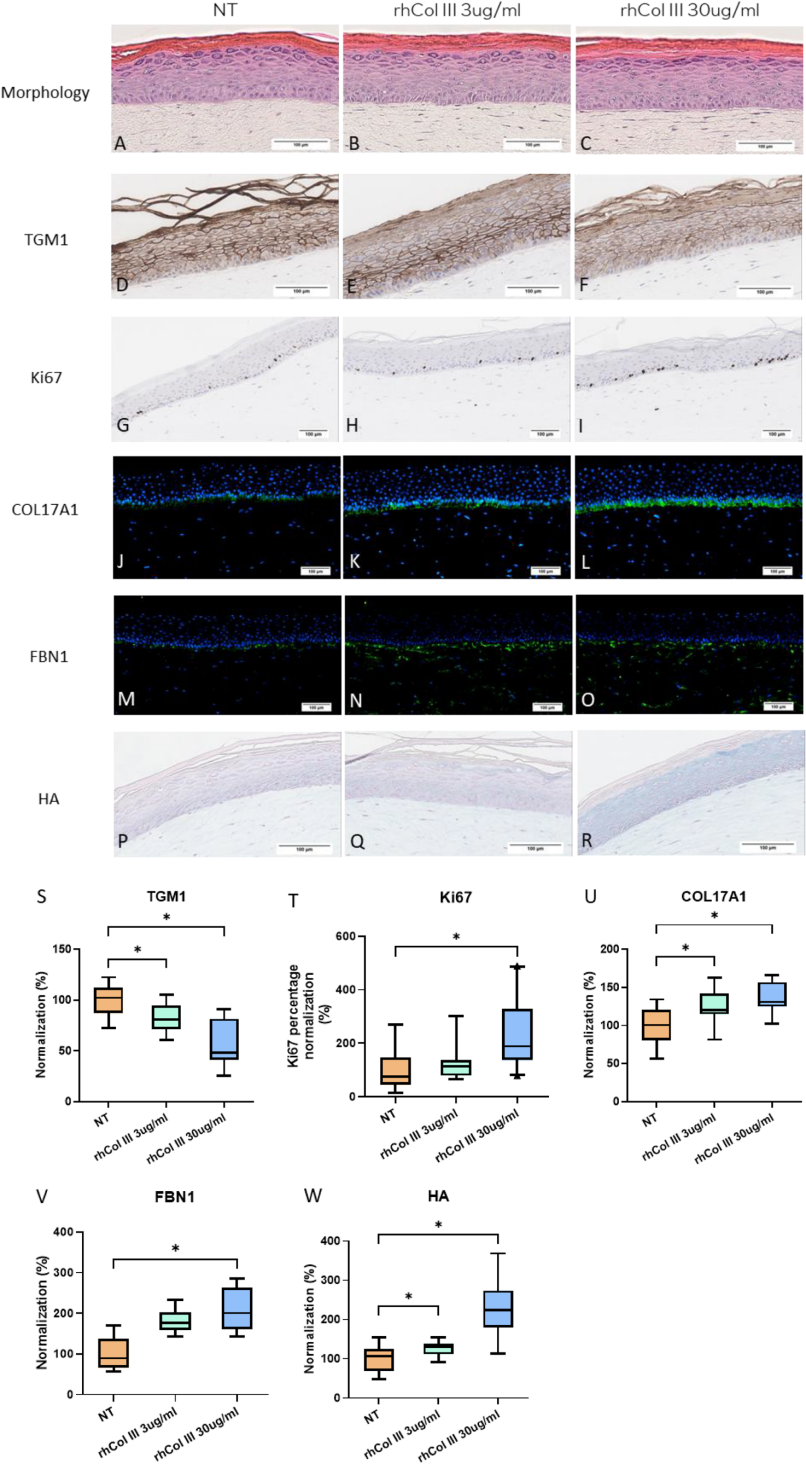
Evaluation of rhCol III peptide effects in reconstructed skin models at two concentrations (3 and 30 μg/mL) for 22 days. (A–C) Histological analysis by H&E staining, which represented the quality of the reconstructed epidermis on D22. (D–I) Immunohistochemical analysis showing the expression of TGM1 & Ki67. (J–O) Immunofluorescence analysis showing the expression of Col17A1 & FBN1. (P–R) Alcian blue staining showing the expression of GAGs. Scale bar 100um. (S–W) Quantification of biomarkers expression on D22. The intensity of the fluorescence or DAB staining of immunolabeled sections was assessed by a semiquantitative method for TGM1, COL17A1, FBN1, and GAGs. For the proliferation marker, Ki67, the percentage of Ki67‐positive cells in the basal membrane was calculated by normalizing to the total number of basal cells. Data of five biomarkers were normalized to media control (NT) in each experiment and presented as mean ± SEM. *N* = 3. One‐way ANOVA was applied with FDR (Benjamini–Hochberg). *p < 0.05. Statistical details are included in Data S2.

Two concentrations of rhCol III peptide (3 and 30 μg/mL), premixed with the collagen solution, were incorporated into the 3D in vitro filler mimetic skin model during reconstruction. Hematoxylin and eosin (H&E) staining revealed no discernible changes in epidermal morphology, including basal layer nucleation, spinous layer thickness, granule formation, stratum corneum thickness, or dermal fibroblast density (data not shown) (Figure 3A–C). Immunohistochemical (IHC) staining for transglutaminase 1 (TGM1), a marker of differentiation/cornification, demonstrated reduced expression with 3 and 30 μg/mL rhCol III peptide (Figure 3D–F). Conversely, IHC staining for Ki67, a marker of basal layer proliferation, revealed an increased proliferation rate at 30 μg/mL rhCol III peptide (Figure 3G,H). Immunofluorescence staining for COL17A1, a marker of epidermal stem cells, showed enhanced expression with both rhCol III peptide supplements (Figure 3L–N). Similarly, FBN1, an ECM component, exhibited increased expression following treatment (Figure 3O–Q). Alcian blue staining, used to visualize glycosaminoglycan distribution, indicated a modest content increase with 3 μg/mL rhCol III peptides and a more pronounced increase with 30 μg/mL, affecting both the epidermal and dermal compartments.

### 3.4 Transcriptomic Implications on Underlying Mechanisms of Actions

To investigate the impact of rhCol III peptide mixture on T‐skin at the gene expression level, RNA‐Seq was performed on 12 samples (3 epidermis control samples, 3 dermis control samples, 3 epidermis samples treated with 30 μg/mL rhCol III peptides, 3 dermis samples treated with 30 μg/mL rhCol III peptides) to assess gene expression profiles across the dermis and epidermis. We acquired a total of 580 908 716 clean reads with an average of 48 409 060 reads (range 39 941 778 to 62 627 264) per sample. Principal component analysis (PCA) revealed significant differences between treatment and control groups in both the dermis and epidermis. Spearman analysis indicated strong concordance within intra‐class samples. These results showed the robustness and reliability of the transcriptomic data, making it suitable for downstream analysis.

**FIGURE 4:**
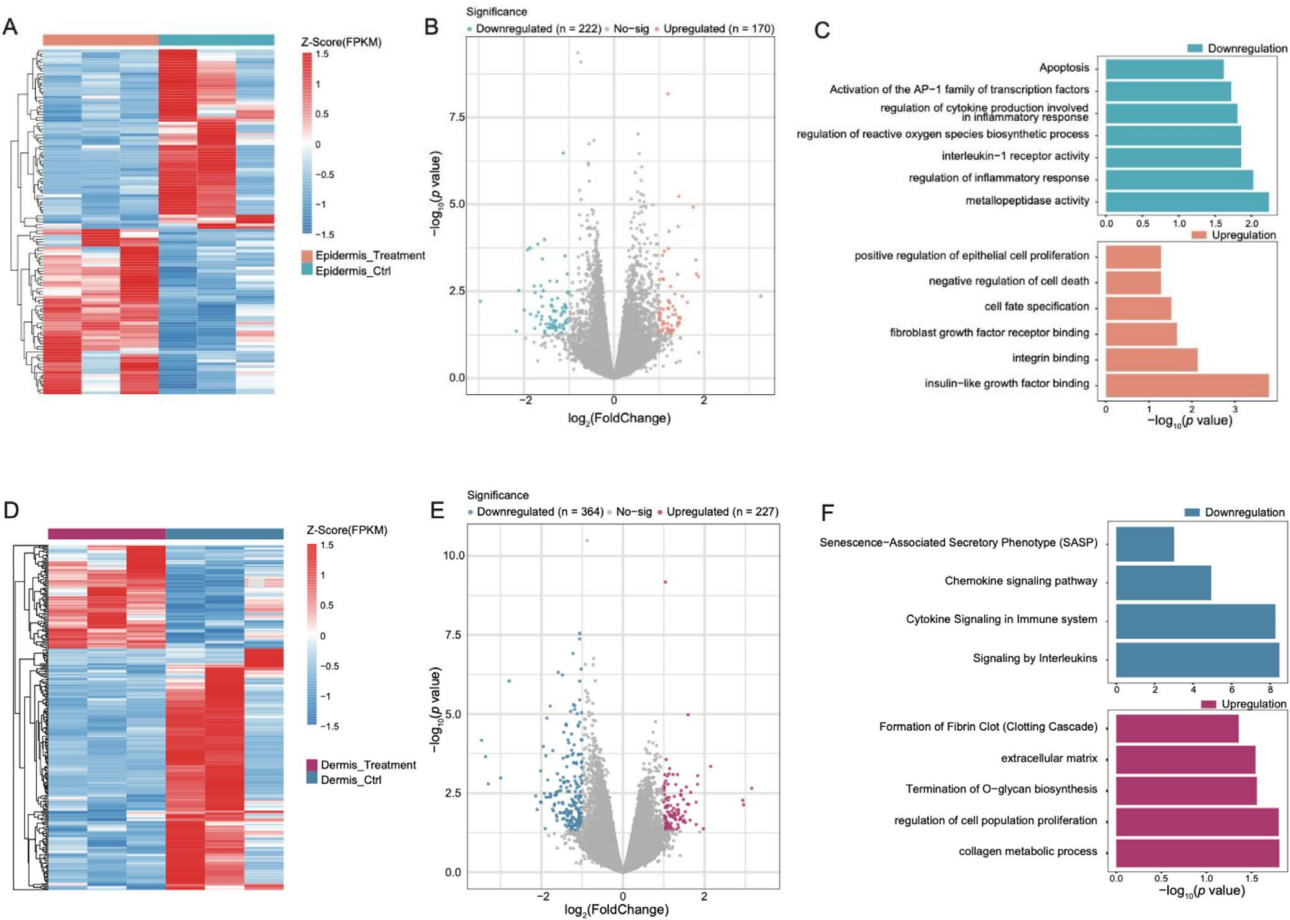
(A) Heatmap of DEGs between epidermis treatment group and epidermis control group. (B) Volcano plots of DEGs in epidermis group (rhCol III peptides 30 μg/mL treatment vs. control). DEGs were screened with a cut‐off criterion of *p* < 0.05 and |log_2_FC| > 1. (C) Bar plots indicating upregulated (orange bar, bottom panel) and downregulated (green bar, top panel) biological pathways in the epidermis group. (D) Heatmap of DEGs between epidermis treatment group and epidermis control group. (E) Volcano plots of DEGs in dermis group (rhCol III peptides 30 μg/mL treatment vs. Control). DEGs were screened with a cut‐off criterion of *p* value < 0.05 and |log_2_FC| > 1. (F) Bar plots indicating upregulated (purple bar, bottom panel) and downregulated (blue bar, top panel) biological pathways in the dermis group.

Therefore, the differentially expressed genes (DEGs) between the treatment and control groups for both dermis and epidermis were calculated with DESeq2 method. In the epidermis, 170 upregulated DEGs were identified (log_2_FC > 1, *p*‐value < 0.05), while 222 DEGs were significantly downregulated (log_2_FC < −1, *p*‐value < 0.05) (Figure 4A,B). Enrichment analysis showed that the biological effects of rhCol III peptides mixture on T‐skin primarily entailed pathways related to insulin‐like growth factor (IGF) binding, integrin binding, fibroblast growth factor (FGF)_receptor binding, cell fate specification, negative regulation of cell death, positive regulation of epithelial cell proliferation. Pathways such as activation of the AP‐1 family of transcription factors, apoptosis, metallopeptidase activity, regulation of inflammatory response, interleukin‐1 receptor activity, regulation of reactive oxygen species biosynthetic process, regulation of cytokine production involved in inflammatory response were significantly downregulated (Figure 4C). In the dermis, 227 DEGs were significantly expressed higher than the control group (log_2_FC > 1, *p*‐value < 0.05), and 364 DEGs were downregulated (log_2_FC < −1, *p*‐value < 0.05) (Figure 4D,E). In response to rhCol III peptides, upregulated DEGs were enriched in pathways such as collagen metabolic process, regulation of cell population proliferation, termination of O‐glycan biosynthesis, extracellular matrix, formation of fibrin clot (Clotting Cascade), the downregulated DEGs were associated with signaling by interleukins, cytokine signaling in immune system, chemokine signaling pathway, and senescence‐associated secretory phenotype (SASP) (Figure 4F). The DEG list with FDR and enrichment tables are provided in Data S3, and key scripts/commands are provided in Data S6.

## 4 Discussion

Collectively, the findings from the present study demonstrated that the treatment with rhCol III peptides mixture induced significant biological changes in T‐skin models. The rhCol III peptides mixture facilitated epidermal cell growth and differentiation, enhanced extracellular matrix formation and collagen synthesis, and suppressed the inflammatory response in both the epidermis and dermis. Consequently, rhCol III peptides emerged as a distinct and promising ingredient for promoting skin regeneration [33, 34].

The nutraceutical benefits and underlying mechanisms of orally administered hydrolyzed collagen peptides have been investigated using serum separated from collagen‐fed rats to treat skin dermal fibroblasts [19]. Certain orally ingested collagen peptides exhibit skin accumulation and demonstrate the ability to counteract decreased fibroblast proliferation and the downregulation of collagen type I and hyaluronan synthesis by modulating the TGF‐β/Smad signaling pathway. Further evidence suggests that animal‐derived collagen peptides stimulate the expression of ECM structural proteins such as collagen, elastin, and versican [18, 35]. The anti‐inflammatory effects of hydrolyzed collagen on skin fibroblasts and keratinocytes, mediated through modulation of IL‐1β, IL‐6, IL‐8, TNF‐α, TGF‐β, and VEGF, have also been reported [36]. Additionally, recombinant human collagen α‐1 peptides, designed based on bioactive domains within the native collagen chain, have demonstrated enhanced collagen and elastin production in human dermal fibroblasts [16]. Based on these findings, we investigated the matrix remodeling effects of rhCol III peptides in human dermal fibroblasts at the gene expression level, focusing on key structural proteins (COL1A1, COL3A1, ELN), adhesive proteins (FBN1), and hyaluronic acid synthases (HAS1‐3). Consistent with previous reports, we observed upregulation of all target genes, albeit with varying effect sizes. Interestingly, the most pronounced effects were observed for genes beyond the core structural proteins COL1A1 and ELN (Figure 1), which are already abundant within the dermal matrix. These results suggest that our rhCol III peptides contribute to matrix remodeling primarily through modulation of adhesive proteins and glycosaminoglycan synthesis rather than by simply increasing the abundance of structural proteins. This mechanism may involve a cascade of events initiated by changes in adhesive protein expression, subsequently influencing the organization and deposition of structural components.

Before investigating the effects within the filler biomimetic 3D in vitro skin model, we addressed key questions regarding the behavior of premixed rhCol III peptides during dermal reconstruction: (1) the extent of rhCol III peptide retention within the dermis compared to the surrounding medium, and (2) the mode of deposition within the derm scaffold to facilitate molecular signaling. A 1 mg/mL aqueous solution of rhCol III peptides was labeled with fluorescein isothiocyanate (FITC) via N‐hydroxysuccinimide (NHS) ester chemistry under mildly alkaline conditions. Following dialysis to remove unbound FITC and excess salts, the FITC‐rhCol III peptides solution was adjusted to 1 mg/mL for the proof‐of‐concept assay (Figure 2). The addition of FITC‐rhCol III peptides to the collagen solution did not affect dermal lattice contraction in terms of shape or diameter (data not shown). Retention of rhCol III peptides within the contracted lattice was roughly calculated based on interpolated concentrations according to a standard curve, disclosing a retention percentage of 71.52% (Data S5). Fluorescence microscopy revealed a distinctly uneven distribution of the FITC signal, with a markedly stronger signal localized within the dermal constructs. This uneven distribution suggests that rhCol III peptides deposition within the dermal scaffold occurs predominantly through an adherent interaction rather than uniform saturation. Combined fluorescence and multiphoton microscopy provided further insights into this deposition pattern. The scaffold‐like distribution of the FITC‐rhCol III peptides signal observed by confocal fluorescence microscopy corresponded precisely with the autofluorescence signal of the derm scaffold captured by multiphoton microscopy. This proof‐of‐concept validation provided a strong rationale for constructing the filler biomimetic skin model and conducting subsequent bioactivity analyses. However, the use of FITC labeling presented limitations. The collagenous composition of the dermal constructs made it challenging to definitively exclude nonspecific binding of free FITC to the scaffold. While the NHS ester linkage formed during FITC labeling is generally considered stable, the possibility of subsequent FITC release due to bond hydrolysis cannot be entirely dismissed. We proceeded under the assumption that the observed FITC signal predominantly represents FITC‐rhCol III peptides bound to the dermal scaffold. However, further investigation using alternative labeling strategies or more rigorous quantification methods may be warranted to confirm this interpretation.

Establishing a dosage rationalization, in alignment with clinical application, is a critical aspect of in vitro experimental design. For instance, a standard clinical injection (e.g., 8 mL of a 2 mg/mL peptide solution applied over an average facial area of 240 cm^2^) provides an estimated clinically relevant dose of 66.7 μg/cm^2^ peptide. Moreover, an in vivo dose of 80 μg/cm^2^ rhCol III peptide injection displayed good performance [27]. Based on these references, dermal premixtures were prepared using a 2 mg/mL rhCol III peptide solution, yielding final concentrations of 3 and 30 μg/mL. Skin morphology was assessed on day 22 post‐rhCol III peptides premixing. No discernible changes in epidermal morphology were observed, including viable epidermal thickness, nucleated cell count, granular layer density, stratum corneum thickness, and density (Figure 3A–C). These observations prompted an examination of epidermal differentiation markers. Immunohistochemical (IHC) staining and analysis of transglutaminase 1 (TGM1) revealed decreased protein expression following rhCol III peptides treatment. Filaggrin (FLG) was also examined by immunofluorescence (IF) staining, displayed no obvious alteration, which indicating no compromise of skin barrier post rhCol III peptides treatment (Data S4). This observation raises the hypothesis that enhanced skin hydration reported after oral hydrolyzed collagen administration may have attributed, in part, to loosened epidermal junctions, facilitating increased transepidermal water loss [8].

Consistent with reports of enhanced cell proliferation in 2D cell culture models, we assessed keratinocyte proliferation within the basal layer of the 3D tissue model. IHC staining for Ki67, a proliferation marker, showed a modest increase in positive cells within the basal layer following rhCol III peptides treatment. The stimulatory effects on matrix adhesive protein (FBN1) and hyaluronic acid synthases (HAS1–3) observed at the gene expression level were also reflected at the 3D tissue level, as demonstrated by immunofluorescence staining and analysis of FBN1 and Alcian blue staining for glycosaminoglycans. To further investigate dermo‐epidermal crosstalk, we examined the impact of rhCol III peptides on epidermal stem cell characteristics. Immunofluorescence staining for COL17A1, an epidermal stem cell marker, revealed increased expression following rhCol III peptides treatment. Notably, all statistically significant effects were observed at the higher, in vivo–relevant rhCol III peptides concentration of 30 μg/mL. These 3D tissue‐level findings not only corroborate the 2D cellular data but also provide valuable insights into the potential mechanisms of action, facilitating a deeper understanding of the molecular pathways involved.

To gain a broader understanding of the regulatory changes induced by rhCol III peptides treatment and to guide further investigation, we performed transcriptomic analysis on the 3D skin models (Figure 4). A 30 μg/mL rhCol III peptide‐treated model was compared to the untreated control. A notable downregulation of inflammation‐related signaling pathways was observed in both the epidermis and dermis, consistent with previous findings in 2D cell culture [15] and in vivo studies [27]. However, due to the absence of immune cells in our current in vitro skin model, we did not observe significant changes in the protein levels of inflammation‐related biomarkers. Nonetheless, the upregulation of cell proliferation and cell fate specification markers (Ki67 and COl17A1, respectively) in the epidermis observed at the protein level was mirrored in the transcriptomic data. Furthermore, the upregulation of fibrin formation and glycan metabolism pathways in the dermis aligns with the increased expression of FBN1 and glycosaminoglycans observed previously (Figure 3). The in vivo bio‐response subsequent to injection is significantly more intricate. While the in vitro findings presented here partially reflected the biological regulation that occurred, they necessitate comprehensive complementation and rigorous reconfirmation in subsequent clinical studies. A limitation of transcriptomics analysis is that differential expression analysis was primarily based on raw *p*‐values rather than FDR‐adjusted values. This approach was adopted because the limited sample size and relatively weak signals resulted in very few genes passing stringent FDR thresholds, which would otherwise preclude downstream biological exploration. We therefore considered the identified genes as exploratory findings and sought to mitigate the risk of false positives by integrating functional enrichment analyses and experimental validation. While this strategy enabled us to generate biologically meaningful hypotheses, we explicitly acknowledge the lack of multiple‐testing correction as a limitation.

It is well‐established that collagen matrices provide an ideal environment for studying fibroblast behavior [37], particularly regarding fibroblast adhesion to the 3D scaffold of bovine collagen and the subsequent cascade of bioactivities triggered by stimulated cytokine secretion. The observed binding of rhCol III peptides to the dermal scaffold likely influences fibroblast adhesion and consequently affects the downstream bioactivities resulting from fibroblast‐secreted cytokines. Therefore, the upregulation of epidermal signaling pathways involving fibroblast growth factor binding, integrin binding, and insulin‐like growth factor binding is noteworthy and warrants further investigation. Still, the lack of immune and vascular components in this 3D scaffold limited the biological finding compared to the comprehensive response from the native skin exposed to true injection.

## Author Contributions

C.H. and R.W. designed the research, performed the studies, analyzed the data, discussed the results, and wrote the paper. Q.Z. analyzed the data, discussed the results, and wrote the paper. Y.W. performed the studies and analyzed the data. N.H. discussed the results and wrote the paper.

## Funding

This study was supported by L'Oreal Research and Innovation Group.

## Ethics Statement

The authors have nothing to report.

## Consent

All authors have read and approved the final manuscript.

## Conflicts of Interest

The authors declare no conflicts of interest.

## Supporting information


**Data S1:** Statistical details of qPCR.

**Data S2:** Statistical details of histology/IF.

**Data S3:** The DEG list with FDR and enrichment tables.

**Data S4:** Immunofluorescence staining and analysis of FLG. (A) Barrier impact of rhCol III peptide in reconstructed skin models at two concentrations (3 and 30 μg/mL) for 22 days. No significant difference was observed among each group. Three tissue replicates/treatment were included in IF staining and the whole tissue section was taken into image analysis by Image J software. (B) Brown‐Forsythe and Welch ANOVA test was applied to all group comparisons with FDR (Benjamini‐Hochberg) correction. Diagrams were generated using GraphPad Prism 10.2.2(397) software (GraphPad Software Inc., La Jolla, CA, USA).

**Data S5:** Retention of rhCol III peptide in dermis. (A) Standard curve of rhCol III peptide. A standard curve was generated with a series of 10‐fold diluted FITC‐rhCol III peptide solutions and corresponding fluorescence intensity. (B) Peptide retention percentage calculation. 210 μg/sample of FITC‐rhCol III peptide was added to reconstruct the lattice. On day 4, medium volume squeezed out by contracted gel was measured, corresponding fluorescence intensity was acquired. Peptide concentrations were interpolated according to the standard curve, quantity in medium and matrix were calculated, respectively. A final rhCol III peptide retention in matrix was perceived at 71.52%.

**Data S6:** Key scripts/commands facilitated in transcriptomics analysis.

**Data S7:** qPCR details of single peak melt curves and cycling curves.

## Data Availability

The data that supports the findings of this study is available on request from the corresponding author.
