# Supplementary material for: In Vitro Bioactivity of a Recombinant Human Collagen Peptide in a Filler Biomimetic Skin Model
Source: J Cosmet Dermatol. 2025 Dec 12;24(12):e70592. doi: 10.1111/jocd.70592 (PMC12699366; doi:10.1111/jocd.70592)
Supplement: Supplementary file 1 — Data S1: Statistical details of qPCR. [file JOCD-24-e70592-s005.docx]

**Supplement 1.** Statistical details of qPCR

Brown-Forsythe and Welch ANOVA was applied FDR (Benjamini-Hochberg) to have comparisons of NT vs treatments, PC vs rhCol III, low dose of rhCol III vs high dose of rhCol III. Individual P value <0.05 represented a statistically significant Discovery (Yes).
