# Supplementary material for: In Vitro Bioactivity of a Recombinant Human Collagen Peptide in a Filler Biomimetic Skin Model
Source: J Cosmet Dermatol. 2025 Dec 12;24(12):e70592. doi: 10.1111/jocd.70592 (PMC12699366; doi:10.1111/jocd.70592)
Supplement: Supplementary file 2 — Data S2: Statistical details of histology/IF. [file JOCD-24-e70592-s006.docx]

**Supplement 2.** Statistical details of histology/IF

Brown-Forsythe and Welch ANOVA was applied FDR (Benjamini-Hochberg) to have comparisons of NT vs treatments. Individual P value <0.05 represented a statistically significant Discovery (Yes).
