# Supplementary material for: In Vitro Bioactivity of a Recombinant Human Collagen Peptide in a Filler Biomimetic Skin Model
Source: J Cosmet Dermatol. 2025 Dec 12;24(12):e70592. doi: 10.1111/jocd.70592 (PMC12699366; doi:10.1111/jocd.70592)

**Supplement 4.** Immunofluorescence staining and analysis of FLG. **A.** Barrier impact of rhCol III peptide in reconstructed skin models at two concentrations (3 μg/ml & 30 μg/ml) for 22 days. No significant difference was observed among each group. Three tissue replicates/treatment were included in IF staining and the whole tissue sections of consistent dimensions were taken into image analysis by Image J software. **B.** Brown-Forsythe and Welch ANOVA test was applied to all group comparisons with FDR (Benjamini-Hochberg) correction. Diagrams were generated using GraphPad Prism 10.2.2(397) software (GraphPad Software Inc., La Jolla, CA, USA).


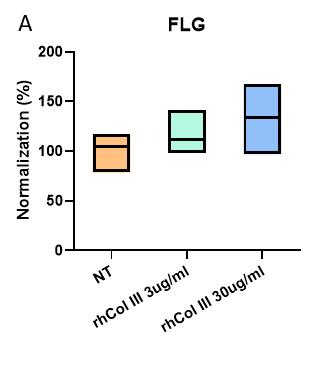


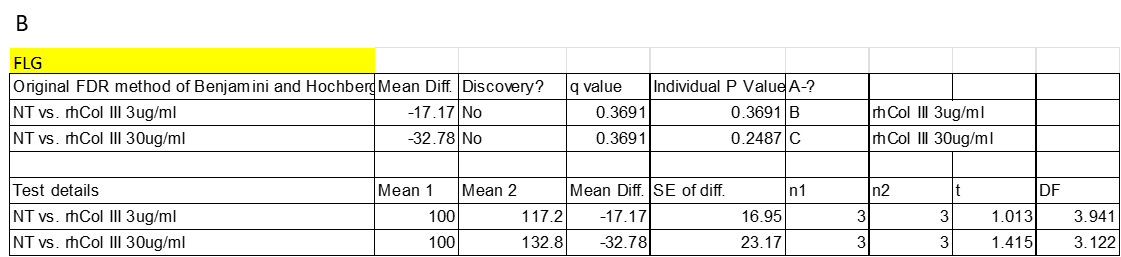

Supplement: Supplementary file 4 — Data S4: Immunofluorescence staining and analysis of FLG. (A) Barrier impact of rhCol III peptide in reconstructed skin models at two concentrations (3 and 30 μg/mL) for 22 days. No significant difference was observed among each group. Three tissue replicates/treatment were included in IF staining and the whole tissue section was taken into image analysis by Image J software. (B) Brown‐Forsythe and Welch ANOVA test was applied to all group comparisons with FDR (Benjamini‐Hochberg) correction. Diagrams were generated using GraphPad Prism 10.2.2(397) software (GraphPad Software Inc., La Jolla, CA, USA). [file JOCD-24-e70592-s001.docx]
