# Supplementary material for: In Vitro Bioactivity of a Recombinant Human Collagen Peptide in a Filler Biomimetic Skin Model
Source: J Cosmet Dermatol. 2025 Dec 12;24(12):e70592. doi: 10.1111/jocd.70592 (PMC12699366; doi:10.1111/jocd.70592)

**Supplement 5.** Retention of rhCol III peptide in dermis. **A.** Standard curve of rhCol III peptide. A standard curve was generated with a series of 10-fold diluted FITC-rhCol III peptide solutions and corresponding fluorescence intensity. **B.** Peptide retention percentage calculation. 210 μg sample of FITC-rhCol III peptide was added to reconstruct the lattice. On day 4, medium volume squeezed out by contracted gel was measured, corresponding fluorescence intensity was acquired. Peptide concentrations were interpolated according to the standard curve, quantity in medium and matrix were calculated, respectively. A final rhCol III peptide retention in matrix was perceived at 71.52%.


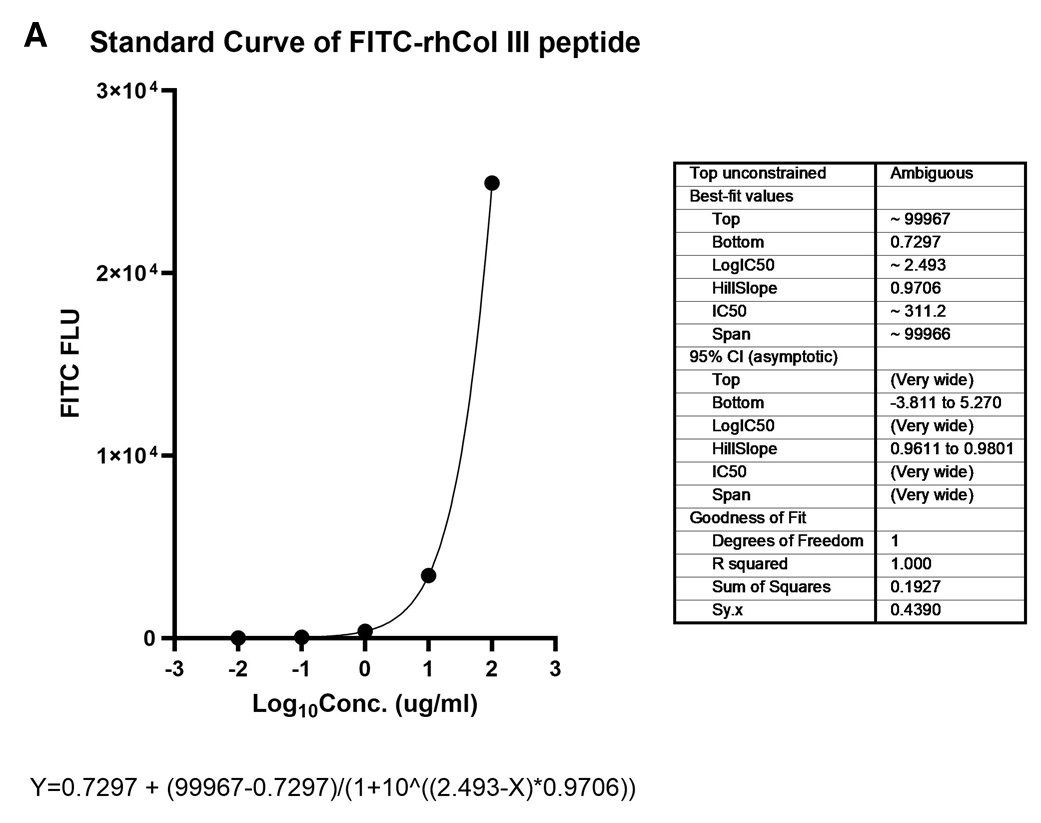


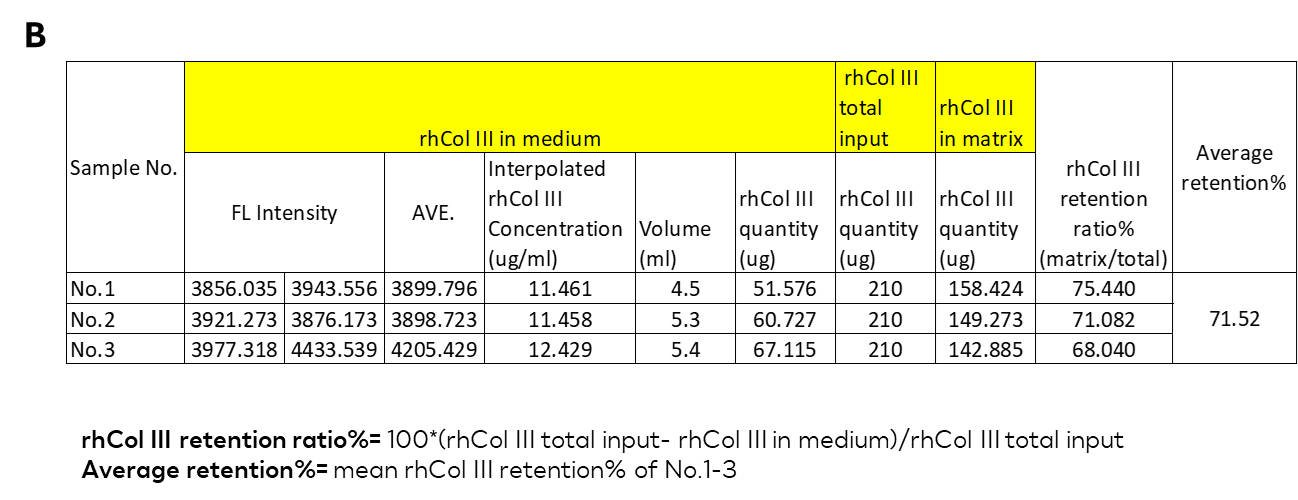

Supplement: Supplementary file 5 — Data S5: Retention of rhCol III peptide in dermis. (A) Standard curve of rhCol III peptide. A standard curve was generated with a series of 10‐fold diluted FITC‐rhCol III peptide solutions and corresponding fluorescence intensity. (B) Peptide retention percentage calculation. 210 μg/sample of FITC‐rhCol III peptide was added to reconstruct the lattice. On day 4, medium volume squeezed out by contracted gel was measured, corresponding fluorescence intensity was acquired. Peptide concentrations were interpolated according to the standard curve, quantity in medium and matrix were calculated, respectively. A final rhCol III peptide retention in matrix was perceived at 71.52%. [file JOCD-24-e70592-s002.docx]
