# Supplementary material for: In Vitro Bioactivity of a Recombinant Human Collagen Peptide in a Filler Biomimetic Skin Model
Source: J Cosmet Dermatol. 2025 Dec 12;24(12):e70592. doi: 10.1111/jocd.70592 (PMC12699366; doi:10.1111/jocd.70592)
Supplement: Supplementary file 6 — Data S6: Key scripts/commands facilitated in transcriptomics analysis. [file JOCD-24-e70592-s003.docx]

#-------Volcano-------

library(ggplot2)

library(dplyr)

result <- read.csv("your_file.csv")

result$Significance <- ifelse(result$pvalue < 0.05 & result$log2FoldChange > 1, "Upregulated",

ifelse(result$pvalue < 0.05 & result$log2FoldChange < -1, "Downregulated", "No-sig"))

ggplot(result, aes(x = log2FoldChange, y = -log10(pvalue))) +

geom_point(aes(color = Significance), alpha = 0.7) +

scale_color_manual(values = c("Upregulated" = "#C6307C", "Downregulated" = "#4991C1", "No-sig" = "gray")) +

labs(title = "Volcano Plot", x = "log2(FoldChange)", y = "-log10(p-value)") +

theme_minimal()

#-------Heatmap-------

library(pheatmap)

library(openxlsx)

matrix <-read.xlsx("4Heatmap.xlsx",sheet = 1, rowNames = T)

matrix=t(scale(t(matrix)))

pheatmap_color = c(

colorRampPalette(c("#1E90FF", "white"))(100),

colorRampPalette(c("white", "red"))(100)

)

range(matrix)

breaks = unique(c(seq(-2, 0, length = 100), 0, seq(0, 2, length = 100)))

annotation_col <- read.xlsx("color.xlsx",sheet = 1, rowNames = T)

ann_colors = list(

group = c(

D_Ctrl = "#4991C1",

D_Treatment = "#C6307C"

)

)

p=pheatmap(

matrix,

cluster_rows = T,

col = pheatmap_color,

breaks=breaks,

annotation_col = annotation_col,

annotation_colors = ann_colors,

fontsize_row = 6,

show_colnames = F

)

#-------Barplot-------

library(ggplot2)

library(patchwork)

library(openxlsx)

df <- read.xlsx("pathway.xlsx",sheet = 1)

df$Term <- reorder(df$Term, df$PValue)

PA = ggplot(df,aes(Term, -log10(PValue)))

PA = PA + geom_bar(aes(fill=Group),stat = "identity")+

coord_flip()+

labs(x='',y='', title = 'Enrichment result')+

scale_fill_manual(values = c("#C6307C")

)

PA

df <- read.xlsx("pathway.xlsx",sheet = 2)

df$Term <- reorder(df$Term, df$PValue)

PB = ggplot(df,aes(Term, -log10(PValue)))

PB = PB + geom_bar(aes(fill=Group),stat = "identity")+

coord_flip()+

labs(x='',y='', title = 'Enrichment result')+

scale_fill_manual(values = c("#4991C1")

)

PB

PB / PA
