# Supplementary figures and images for: In Vitro Bioactivity of a Recombinant Human Collagen Peptide in a Filler Biomimetic Skin Model
Source: J Cosmet Dermatol. 2025 Dec 12;24(12):e70592. doi: 10.1111/jocd.70592 (PMC12699366; doi:10.1111/jocd.70592)

**Supplement 7.** qPCR details of single peak melting curves and cycling curves.


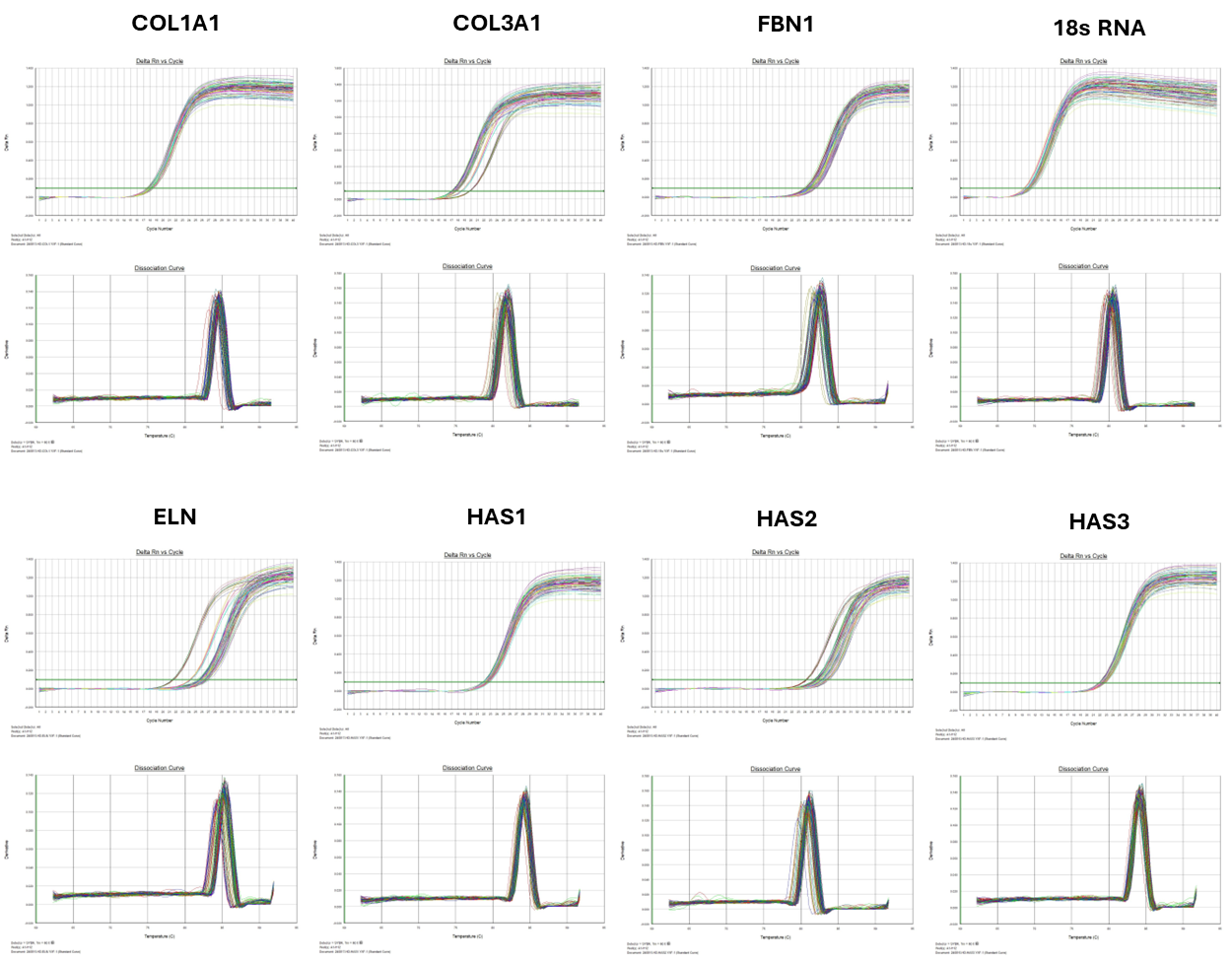

Supplement: Supplementary file 7 — Data S7: qPCR details of single peak melt curves and cycling curves. [file JOCD-24-e70592-s004.docx]
